# Non-occlusive mesenteric ischemia during acute stroke management: three case reports

**DOI:** 10.1186/s40792-022-01392-y

**Published:** 2022-02-28

**Authors:** Kazuki Fujii, Tadahiko Seki, Yasuyuki Nakata, Kazuaki Atagi, Takeshi Matsuyama

**Affiliations:** Nara Prefecture General Medical Center, 2-897-5, Schichijo Nishimachi, Nara, Nara 630-8581 Japan

**Keywords:** Non-occlusive mesenteric ischemia, Enteral nutrition, Stroke

## Abstract

**Background:**

There are many reports of non-occlusive mesenteric ischemia in patients on maintenance hemodialysis and following cardiac surgery. However, there are few reports of non-occlusive mesenteric ischemia in patients with acute stroke.

**Case presentation:**

We report three cases of non-occlusive mesenteric ischemia with onset during treatment for acute stroke. All of the patients were undergoing strict blood-pressure control, and two patients developed NOMI soon after tracheostomy when enteral nutrition had been resumed.

**Conclusion:**

Many stroke patients are older adults with risk factors such as arteriosclerosis. Thus, during acute stroke management, there is a possibility that patients may develop non-occlusive mesenteric ischemia due to decreased intestinal blood flow secondary to strict blood-pressure control. This case report implicates early enteral nutrition as a potential etiopathogenic factor of non-occlusive mesenteric ischemia in patients with acute stroke.

## Background

Non-occlusive mesenteric ischemia (NOMI) is an acute mesenteric circulatory disorder that occurs without organic vascular occlusion and is associated with extremely high mortality [1]. Potential risk factors for NOMI include age, renal disease, diabetes mellitus, reduced cardiac output, vasopressor use, intra-aortic balloon pump, and increased inflammatory markers [2–4]. However, there are few reports of NOMI in acute stroke. This report aims to present the key features of NOMI in patients with acute stroke.

## Case presentation

Among the 296 stroke patients (110, 103, and 83 patients with cerebral infarction, subarachnoid hemorrhage, and intracerebral hemorrhage, respectively) treated in the intensive care unit (ICU) of our hospital from May 2018 to January 2021, based on contrast-enhanced computed tomography (CECT) scans of the abdomen, we identified three patients with acute stroke and NOMI.

### Case 1

**Fig. 1:**
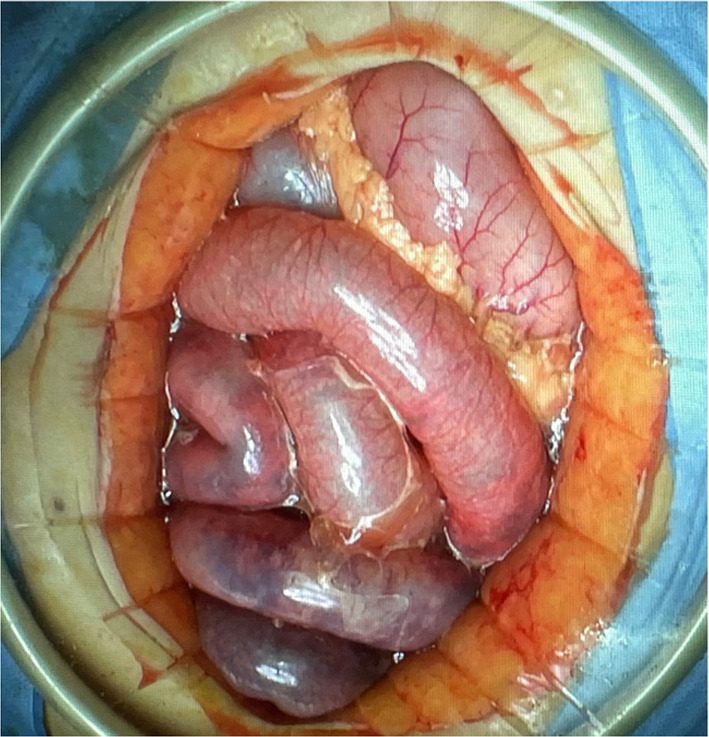
Intraoperative image. Small intestinal and ascending colon necrosis with skip lesions was revealed in emergency laparotomy

A 60-year-old woman underwent coil embolization for subarachnoid hemorrhage due to a basilar artery aneurysm rupture, but developed abdominal distension, vomiting, and metabolic acidosis on the sixth postoperative day. Abdominal CECT showed widespread contrast defects in the small intestine along with portal and intestinal emphysema, which indicated NOMI. Emergency laparotomy was performed on the same day and revealed small intestinal and ascending colon necrosis with skip lesions (Fig. 1). Indocyanine green (ICG) fluorography to evaluate intestinal blood flow showed poor fluorescence emission in the ischemic area of the bowel. The necrotic bowel was resected, but bowel anastomosis or stoma creation was not undertaken. In the resected specimens, histopathology showed massive ischemic changes that reflected discontinuous necrosis of the small bowel and ascending colon without arterial or venous thrombus, which supported a diagnosis of NOMI. The patient was discharged on day 119 of hospitalization.

### Case 2

A 78-year-old woman underwent a craniotomy for subcortical hemorrhage in the right parietal lobe. A tracheostomy was performed on postoperative day 6, and enteral nutrition was started on the same day. To evaluate abdominal distension, a CECT was performed on postoperative day 8 and showed poor contrast uptake in the small intestine and portal emphysema. Thus, NOMI was suspected and, on the same day, emergency laparotomy revealed small bowel and ascending colon necrosis with skip lesions. ICG fluorography to evaluate intestinal blood flow revealed the ischemic bowel segments as regions of poor fluorescence emission. The necrotic bowel was resected. Bowel anastomosis was not performed, and a stoma was created. Histopathological findings supported a diagnosis of NOMI, and the patient was discharged on day 35 of hospitalization.

### Case 3

An 82-year-old woman underwent coil embolization for subarachnoid hemorrhage due to internal carotid artery–posterior communicating artery aneurysmal rupture. Enteral nutrition was started on postoperative day 2, and a tracheostomy was performed on postoperative day 7; enteral nutrition was restarted on the same day. CECT for abdominal distension on postoperative day 9 revealed extensive areas with poor contrast uptake in the small intestine and portal emphysema, which indicated NOMI, and an emergency laparotomy that was performed on the same day showed extensive small bowel and colon necrosis with skip lesions. The necrotic bowel was resected, but bowel anastomosis was not performed, and a stoma was created. However, histopathology showed no specific findings, and the patient died of cerebral herniation 16 days after hospitalization. The results are summarized in Table 1.

**Table 1:** Clinical features of patients with NOMI during treatment for acute stroke

	Case 1	Case 2	Case 3
Age (years)	60	78	82
Sex	Female	Female	Female
Stroke	SAH	Subcortical hemorrhage	SAH
Stroke treatment	Coil embolization Antihypertensive therapy	Craniotomy for removal of hematoma antihypertensive therapy	Coil embolization antihypertensive therapy
Risk factors for NOMI	Hypertension	OMI	Hypertension Diabetes mellitus
Treatment for NOMI	Small bowel resection, Right hemicolectomy, stoma creation	Small bowel resection, stoma creation	Small bowel resection, subtotal colectomy, stoma creation
Period from stroke onset to NOMI (days)	7	8	9
Period from tracheostomy onset to NOMI (days)	N/A	2	2
Outcome	Survive	Survive	Death

## Discussion

NOMI causes segmental, discontinuous intestinal vascular obstruction without organic occlusion of the mesenteric vascular trunk [1]. NOMI generally affects patients older than 50 years suffering from myocardial infarction, congestive heart failure, aortic insufficiency, and renal or postoperatively following cardiac surgery [5]. Only one report has discussed NOMI occurring during treatment for acute stroke [6].

The American Society for Parenteral and Enteral Nutrition and Society of Critical Care Medicine 2009 recommendations for early initiation of enteral nutrition [7] have promoted early enteral nutrition for critically ill patients worldwide. Accordingly, stroke patients have undeniably benefited from early enteral nutrition in many ways.

However, NOMI is a disadvantage of early enteral nutrition. Although early enteral nutrition is cautiously administered in patients with known risk factors, enteral nutrition is aggressively used in stroke patients. Given the increasing frequency of NOMI in stroke patients (including suspected cases and cases not treated surgically), we evaluated whether, due to several factors, stroke patients might be at higher risk for NOMI.

One of the pathogenetic mechanisms of NOMI is a mismatch in the oxygen demand-and-supply ratio in the intestine, particularly in the vulnerable superficial mucosa, that is induced by early enteral nutrition [8, 9]. Thus, early enteral nutrition, which is often started earlier in stroke patients because they do not have organic bowel problems, should be recognized as a risk factor for NOMI. Furthermore, for various reasons, stroke patients undergo tracheostomy, followed by early enteral nutrition.

We consider it is preferable to start enteral nutrition after tracheostomy with a small amount or the day after tracheostomy. Of course, pathological condition leading to tracheostomy has greater effect on intestinal peristalsis. Although tracheostomy does not directly affect intestinal movement, we think it does have an indirect effect on decreased intestinal movement. Under such circumstances, early enteral nutrition may be a risk factor for NOMI.

In addition, nicardipine, which is used for strict blood-pressure control, has an inhibitory effect on the gastrointestinal smooth muscle and may cause paralytic ileus (which is a well-known side effect that tends to be ignored). Moreover, administering early enteral nutrition in ileus may lead to bacterial overgrowth and progressive edema, thereby impairing intestinal mucosal blood supply and causing ischemic injury [10].

Many stroke patients have high vascular risks, and optimal intestinal blood flow may not be maintained during strict blood-pressure control. All of the above-mentioned cases occurred under strict blood-pressure control, and two patients developed NOMI early after tracheostomy following resumption of enteral nutrition.

For the above-mentioned reasons, patients with acute stroke should be considered to be at a high risk for NOMI.

## Conclusion

Early enteral nutrition, in addition to its manifold benefits, poses a risk of NOMI.

The stroke patients under strict blood-pressure control may be at increased risk for NOMI, and we should especially be aware when initiating enteral nutrition.

## Data Availability

All data generated or analyzed during this study are included in this published article.

## Abbreviations

NOMI: Non-occlusive mesenteric ischemia
SAH: Subarachnoid hemorrhage
OMI: Old myocardial infarction
